# White Matter Tract Integrity in Alzheimer's Disease vs. Late Onset Bipolar Disorder and Its Correlation with Systemic Inflammation and Oxidative Stress Biomarkers

**DOI:** 10.3389/fnagi.2017.00179

**Published:** 2017-06-16

**Authors:** Ariadna Besga, Darya Chyzhyk, Itxaso Gonzalez-Ortega, Jon Echeveste, Marina Graña-Lecuona, Manuel Graña, Ana Gonzalez-Pinto

**Affiliations:** ^1^Centre for Biomedical Research Network on Mental HealthSpain; ^2^Department of Internal Medicine of Hospital Universitario de AlavaVitoria, Spain; ^3^Computational Intelligence Group, University of the Basque Country (UPV/EHU)San Sebastian, Spain; ^4^ACPySSSan Sebastian, Spain; ^5^Department of Psychiatry, University Hospital of Alava-SantiagoVitoria, Spain; ^6^School of Psychology, University of the Basque Country (UPV/EHU)San Sebastian, Spain; ^7^Magnetic Resonance Imaging DepartmentOsatek, Vitoria, Spain; ^8^School of Medicine, University of the Basque Country (UPV/EHU)Vitoria, Spain

**Keywords:** late onset bipolar disorder, tract based spatial statistics, Alzheimer disease, inflammatory biomarkers, multimodal brain data analysis, nerve growth factors

## Abstract

**Background:** Late Onset Bipolar Disorder (LOBD) is the development of Bipolar Disorder (BD) at an age above 50 years old. It is often difficult to differentiate from other aging dementias, such as Alzheimer's Disease (AD), because they share cognitive and behavioral impairment symptoms.

**Objectives:** We look for WM tract voxel clusters showing significant differences when comparing of AD vs. LOBD, and its correlations with systemic blood plasma biomarkers (inflammatory, neurotrophic factors, and oxidative stress).

**Materials:** A sample of healthy controls (HC) (*n* = 19), AD patients (*n* = 35), and LOBD patients (*n* = 24) was recruited at the Alava University Hospital. Blood plasma samples were obtained at recruitment time and analyzed to extract the inflammatory, oxidative stress, and neurotrophic factors. Several modalities of MRI were acquired for each subject,

**Methods:** Fractional anisotropy (FA) coefficients are obtained from diffusion weighted imaging (DWI). Tract based spatial statistics (TBSS) finds FA skeleton clusters of WM tract voxels showing significant differences for all possible contrasts between HC, AD, and LOBD. An ANOVA *F*-test over all contrasts is carried out. Results of *F*-test are used to mask TBSS detected clusters for the AD > LOBD and LOBD > AD contrast to select the image clusters used for correlation analysis. Finally, Pearson's correlation coefficients between FA values at cluster sites and systemic blood plasma biomarker values are computed.

**Results:** The TBSS contrasts with by ANOVA *F*-test has identified strongly significant clusters in the forceps minor, inferior longitudinal fasciculus, inferior fronto-occipital fasciculus, and cingulum gyrus. The correlation analysis of these tract clusters found strong negative correlation of AD with the nerve growth factor (NGF) and brain derived neurotrophic factor (BDNF) blood biomarkers. Negative correlation of AD and positive correlation of LOBD with inflammation biomarker IL6 was also found.

**Conclusion:** TBSS voxel clusters tract atlas localizations are consistent with greater behavioral impairment and mood disorders in LOBD than in AD. Correlation analysis confirms that neurotrophic factors (i.e., NGF, BDNF) play a great role in AD while are absent in LOBD pathophysiology. Also, correlation results of IL1 and IL6 suggest stronger inflammatory effects in LOBD than in AD.

## Introduction

Bipolar disorder (BD) is a chronic mood disorder characterized by maniac and depressive alternating episodes, interspersed by euthymic periods. Age of onset may be determined by environmental and genetic conditions (Bauer M. et al., 2014; Martinez-Cengotitabengoa et al., 2014; Bauer et al., 2015a,b). Commonly, BD onset happens during youth years, leading to cognitive, affective, and functional impairment (Forcada et al., 2015). When the onset age is above 50 years, it is considered a late onset BD (LOBD) (Depp and Jeste, 2004; Zanetti et al., 2007; Prabhakar and Balon, 2010; Besga et al., 2011; Carlino et al., 2013; Chou et al., 2015), which may be difficult to differentiate from Alzheimer's disease (AD), because of overlapping symptoms (Zahodne et al., 2015). Another example of the fuzzy boundaries between brain pathologies is the discovery of an AD biomarker signature that also identifies Parkinson's Disease patients with dementia (PDD) (Berlyand et al., 2016) opening the door for crossover treatment of PDD with AD therapies. This trend is appreciated in recent studies comparing BD and AD patients (Berridge, 2013). Specifically, inflammation and oxidative stress biomarkers have been identified for AD (Akiyama et al., 2000; Kamer et al., 2008; Sardi et al., 2011), LOBD (Goldstein et al., 2009; Konradi et al., 2012; Leboyer et al., 2012; Lee et al., 2013; Bauer I. E. et al., 2014; Hope et al., 2015), depression, and mania (Brydon et al., 2009; Dickerson et al., 2013; Castanon et al., 2014; Singhal et al., 2014). Common traits between LOBD and AD are described in Besga et al. (2015). Common psychiatric symptoms in AD which are shared with the profile observed in LOBD patients are: agitation, euphoria, disinhibition, over-activity without agitation, aggression, affective liability, dysphoria, apathy, impaired self-regulation, and psychosis (Albert and Blacker, 2006; Zahodne et al., 2015).

This paper contains a new contribution to a comparative study of AD vs. LOBD patients that has been carried out for some time. In this study, demographic and other data gathered from the patients at recruitment, such as psychological tests and MRI data, has been described in Besga et al. (2012), Graña et al. (2011), and Besga et al. (2015, 2016), therefore description of materials can not be duplicated here without breaking imposed journal self-plagiarism rules. Consequently we refer the reader to these publications, while here we provide a summary account of the study and results achieved and reported in previous publications. Over one hundred subjects older than 64 years were recruited, including healthy controls (HC), and AD and LOBD patients. These subjects were treated to neuropsychological tests, blood extraction for plasma biomarkers measurement, and the acquisition of several modalities of magnetic resonance imaging (MRI). Specifically, Diffusion-Weighted Imaging (DWI) was acquired in order to study significant differences in the white matter (WM) structure. Reasons for eligibility and discarding of patients and full account of the materials are given in Besga et al. (2012), Graña et al. (2011), and Besga et al. (2015, 2016), and we dare not reproduced them here. Previously reported results of this study have been the following ones:

1. We demonstrate good discrimination between AD and LOBD populations using whole brain fractional anisotropy (FA) coefficients extracted from the DWI data (Graña et al., 2011; Besga et al., 2012), using multivariate machine learning for computer aided diagnosis (CAD) system design (Sigut et al., 2007; Salas-Gonzalez et al., 2009; Ramirez et al., 2010; Savio et al., 2011; Westman et al., 2011; Termenon et al., 2013). Though the classification performance results were good, the localization of effects in brain regions was not as satisfactory due to the feature extraction process.
2. We achieved encouraging classification results based on the clinical, neuropsychological test, and blood plasma biomarkers (a subset of the ones used in the this paper Besga et al., 2015) using machine learning techniques. We found that clinical variables have the greatest discriminant power. Blood plasma biomarkers alone have little discriminant power but help improve the clinical variables. Besides, we had no anatomical correspondences of the findings because no imaging data was involved.
3. Looking for inferences about the anatomical correlations of blood biomarkers we applied canonical correlation between them and whole brain FA data (Besga et al., 2016) using eigenanatomy tools that decompose the FA volume into eigenvolumes maximally correlated with plasma biomarkers (Avants et al., 2012, 2014) for feature extraction and ensuing AD vs. LOBD classification by machine learning. We found positive correlations of the oxido-nitrosative stress biomarker malondialdehyde (MDA) (Besga et al., 2016) with voxel clusters in the superior corona radiata, internal capsule, and superior longitudinal fasciculus. However, confirmation by classification performance was moderate, accuracy was below 80%.

Besides the commented limitations of previous works motivating further research, measurements of two new inflammation biomarkers were made available for new computational explorations.

### Contributions of this paper

The process carried out in this paper has two phases: first, cluster selection in FA volumes, and, second, a correlation analysis between image clusters and blood biomarkers. For cluster selection, we apply Tract-Based Spatial Statistics (TBSS) (Smith et al., 2006; Bach et al., 2014) to find significant WM tract differences between AD and LOBD patients. We expect TBSS to provide tract specific effects, improving the anatomical finding reported by our previous approaches (Graña et al., 2011; Besga et al., 2012, 2016). TBSS identifies WM tract voxel clusters with significant difference in FA between AD and LOBD on the mean FA skeleton. To enhance localization power we carry out an ANOVA *F*-test. The correlation analysis is carried out by computing Pearson's correlation coffiencients of the FA values at voxel sites and blood biomarker values across all subjects. Machine learning is not used because of the small sample size that makes cross-validation results very unstable and not significant.

## Methods

### Ethics statement

The ethics committee of the Alava University Hospital, Spain, approved this study. All patients gave their written consent to participate in the study, which was conducted according to the provisions of the Helsinki declaration. After written informed consent was obtained, venous blood samples (10 mL) were collected from the volunteers, after which all the mood scales and cognitive tests were performed. The study has been registered as an observation trial^1^ in the ISRCTN registry.

### Blood plasma biomarkers

The blood plasma biomarkers selected for analysis include:

- Neurotrophins: nerve growth factor (NGF) and brain-derived neurotrophic factor (BDNF).
- Inflammation biomarkers (Akiyama et al., 2000; Kamer et al., 2008; Goldstein et al., 2009; Sardi et al., 2011; Lee et al., 2013; Garcia-Bueno and et al., 2014): Cytokines Interleukins 1 and 6 (IL-1β, IL-6) and Tumor Necrosis Factor (TNFα), and the Cyclooxygenases (COX-1 and COX-2) by-products Prostaglandin E_2_ (PGE2) and 15d-Prostaglandin J_2_ (PGJ2).
- Oxidative stress biomarkers: nitrites (NO_2_) and malondialdehyde (MDA).

Their measurements are described in Besga et al. (2015), except for PGE2 and PGJ2 which were measured by enzyme immunoassay (EIA) using reagents in kit form (Prostaglandin E2 EIA Kit-Monoclonal; Cayman Chemical Europe, Tallinn, Estonia and 15-deoxy-Δ12,14- Prostaglandin J2 ELISA Kit DRG Diagnostics, Marburg, Germany). Samples were measured following manufacturer's instructions.

### Diffusion weighted imaging

Diffusion-Weighted Imaging (DWI) uses MRI acquisition sequences computing signal differences along several gradient directions in order to obtain a signal that measures water diffusion. Diffusion Tensor Imaging (DTI) is a compact representation by means of 3 × 3 matrix of water diffusion in each spatial direction at each voxel (Basser et al., 1994; Pierpaoli et al., 1996). Specifically, in this paper we will work on FA values, which are computed as follows:

(1) $$FA\left(j\right)=\;\sqrt{\frac{3\;\sum_{i=1}^{3}{\left(\lambda_{i}-\bar{\lambda }\right)}^{2}}{2\;\sum_{i=1}^{3}\lambda_{i}^{2}}},$$

where {λ_1_, λ_2_, λ_3_} are the diffusion tensor eigenvalues at the voxel site *j*. The specific parameters of the data capture on a 1.5 Tesla scanner (Magnetom Avanto, Siemens), data preprocessing, computing FA, and registration have been given in Besga et al. (2016). We use the FSL software suite (http://www.fmrib.ox.ac.uk/fsl/) to carry out DWI preprocessing, DTI estimation (Behrens et al., 2003), image registration (Andersson et al., 2007a,b), and TBSS described below. We did not perform spatial smoothing. The pre-processing consists in the removal of non-brain voxels using the brain extraction tool (BET) from FSL, the correction of eddy currents artifacts, and the rigid registration of the gradient images to cope with motion of the subject. On the spatially aligned DWI we estimate the diffusion tensor at each voxel, and the FA values. The FA volumes are then spatially normalized by non-linear registration to the FMRIB58_FA template provided with FSL standards. We have not computed a template from the actual FA dataset because the population is small and very heterogenous so the resulting mean FA template is quite noisy and blurry. Besides we need to register the data to the MNI152 space in order to report atlas based localizations, which is already done in the template provided by FSL. We do not carry out any intensity normalization on the FA images.

### TBSS

We apply TBSS (Smith et al., 2006; Bach et al., 2014), a module of FSL (Smith et al., 2004)^2^ to detect differences in white matter tracts between HC, AD, and LOBD subjects. The specific TBSS procedure applied is as follows: (1) We warp the FA volumes according to the registration carried out before, so we have all aligned in the common space. (2) We compute the mean FA image and extract the common skeleton from it by morphological image processing. This skeleton is assumed to represent the centerline of the WM tracts in all the FA volumes. Each subject's aligned FA data is then projected onto this skeleton. This projection is achieved by assigning the closest local maximum of FA value in the orthogonal direction to the skeleton. (3) For each possible contrast (i.e., HC > AD, AD > LOBD, HC > LOBD, AD > HC, LOBD > AD, LOBD > HC, and ANOVA *F*-test over all pairwise contrasts) we compute a permutation test applying the randomize tool of FSL with 50,000 permutations and threshold free cluster enhancement (TFCE)(Smith and Nichols, 2009) skeleton cluster selection over the contrast statistics. The advantage of TFCE over other methods is that it combines the spatial and statistics value optimally achieving high significance, and its parameters are already optimized by an automated procedure.

### Biomarker correlation analysis

The correlation analysis is applied to the clusters selected by each binary contrast masked by the *F*-test cluster detection. The resulting clusters are much smaller than the binary contrast detection but more specific. Considering independently each voxel site *j* of the selected clusters, we build a vector **v**_*j*_ composed of the FA intensities at the *j*-th voxel site across all the subjects. We compute Pearson's correlation coefficient between this vector and the value of the blood biomarker for this subject, denoted *y*_*i*_ in the following, obtaining the correlation values at each voxel site. Pearson's correlation (Pearson, 1895; Kendall and Stuart, 1973) at the *j*-th voxel site is computed as follows:

(2) $$r_{v_{j},y}=\frac{n\sum_{i}v_{ij}y_{i}-\sum_{i}v_{ij}\sum_{i}y_{i}}{\sqrt{n\sum_{i}v_{ij}^{2}-{\left(\sum_{i}v_{ij}\right)}^{2}}\sqrt{n\sum_{i}y_{i}^{2}-{\left(\sum_{i}y_{i}\right)}^{2}}},$$

where *v*_*ij*_ is the value of FA at the *j*-th voxel site in the*i*-th subject, and *y*_*i*_ is the blood plasma biomarker value of that *i*-th subject. We compute correlation values for each subpopulation (i.e., AD and LOBD) independently (following a reviewer recommendation), retaining voxels significantly correlated (*p* < 0.01) for examination.

### Atlas based effect localization

Locations reported by atlasquery tool from FSL using the JHU White-Matter Tractography Atlas are collected for each contrast after the permutation test, and each correlation analysis between detected FA skeleton clusters and blood biomarkers. The report produced by the atlasquery tool are the probability of a cluster voxel to belong to a tract, therefore the size of the cluster falling in a tract is computed as product of atlasquery probabilities and the total detection volume.

## Results

**Figure 1:**
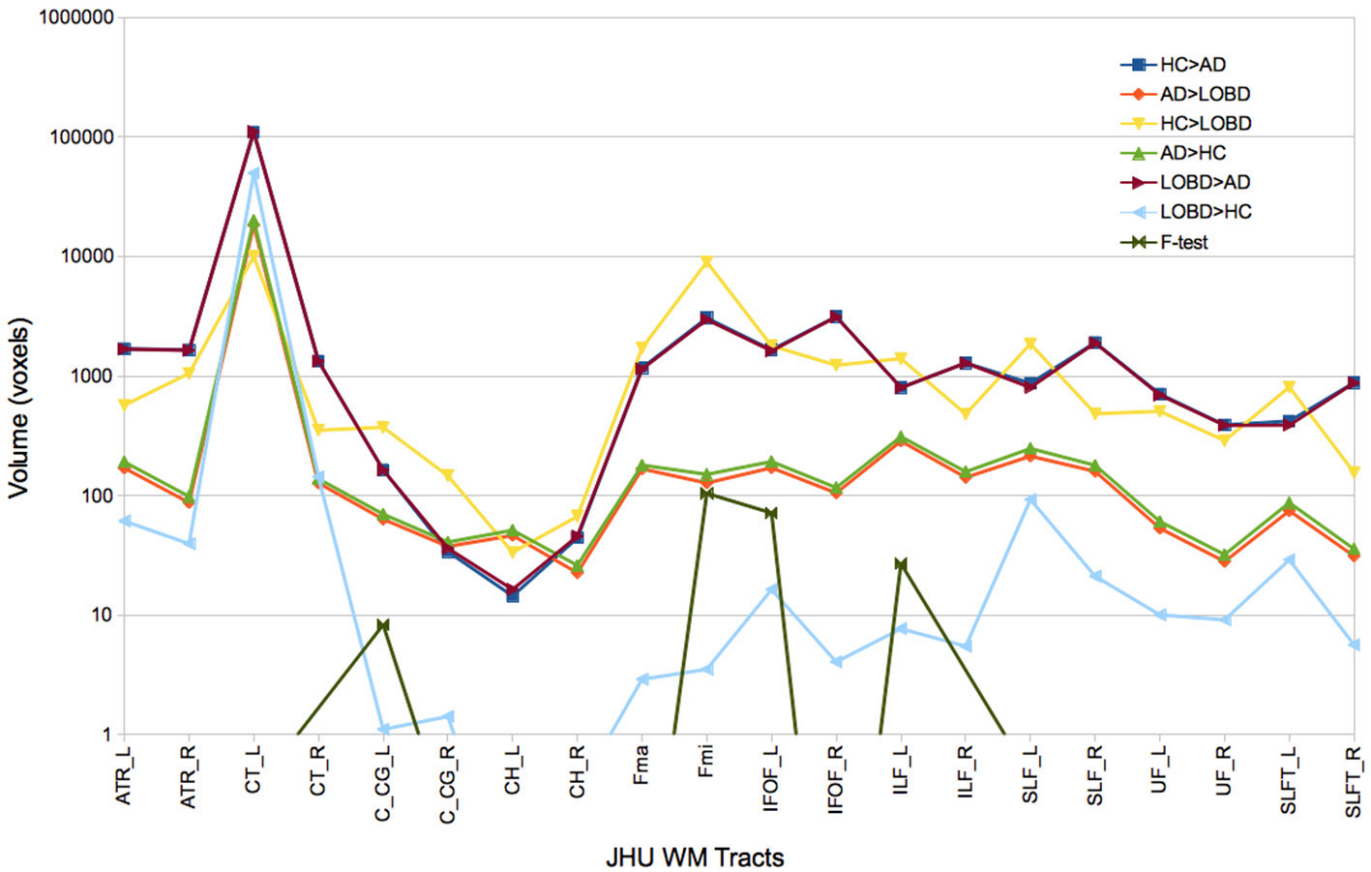
Size of FA skeleton clusters found by each contrast (HC > AD, AD > LOBD, HC > LOBD, AD > HC, LOBD > AD,LOBD > HC, *F*-test) of the permutation test followed by TFCE cluster inference for each tract identified by the JHU White-Matter Tractography Atlas. R, Right; L, left; ATR, hemispheres of anterior thalamic radiation; CT, corticospinal tract; C_CG, cingulum (cingulate gyrus); CH, Cingulum (hippocampus); FMi, forceps minor; FMa, forceps major; IFOF, inferior fronto-occipital fasciculus; ILF, inferior longitudinal fasciculus; SFL, superior longitudinal fasciculus; UF, uncinate fasciculus; SLFT, temporal part of SLF.

Figure 1 plots the size (logarithmic scale) of the skeleton clusters found by the permutation test for each of the contrasts including the *F*-test for each tract that can be identified in the JHU White-Matter Tractography Atlas, i.e., Right (R) and left (L) hemispheres of anterior thalamic radiation (ATR), corticospinal tract (CT), cingulum (cingulate gyrus) (C_CG), Cingulum (hippocampus) (CH), forceps minor (FMi), forceps major (FMa), inferior fronto-occipital fasciculus (IFOF), inferior longitudinal fasciculus (ILF), superior longitudinal fasciculus (SFL), uncinate fasciculus (UF), temporal part of SLF (SLFT). Before masking with *F*-test results, pairwise contrasts greatest effects are located in the CT, ATR, FMa, and Fmi. However big, CT clusters are of similar size for all contrast, and disappear after *F*-test masking. Similarly, ATR clusters are reduced while ILF clusters relative importance increase after *F*-test masking. Clusters where HC or LOBD have greater FA signal than AD (HC > AD, LOBD > AD) are much bigger (note log scale in the plot) across all the tracts than the converse (AD > HC, AD > LOBD). Also, clusters of HC > LOBD are much bigger than LOBD > HC. These differences in effect size are strongly significant (*p* < 0.00001, pairwise *t*-tests) The *F*-test selection involves mostly the C_CG, Fmi, IFOF, and ILF tracts. *F*-test detections in other tract is marginal, though we have included them in the correlation analysis.

**Figure 2:**
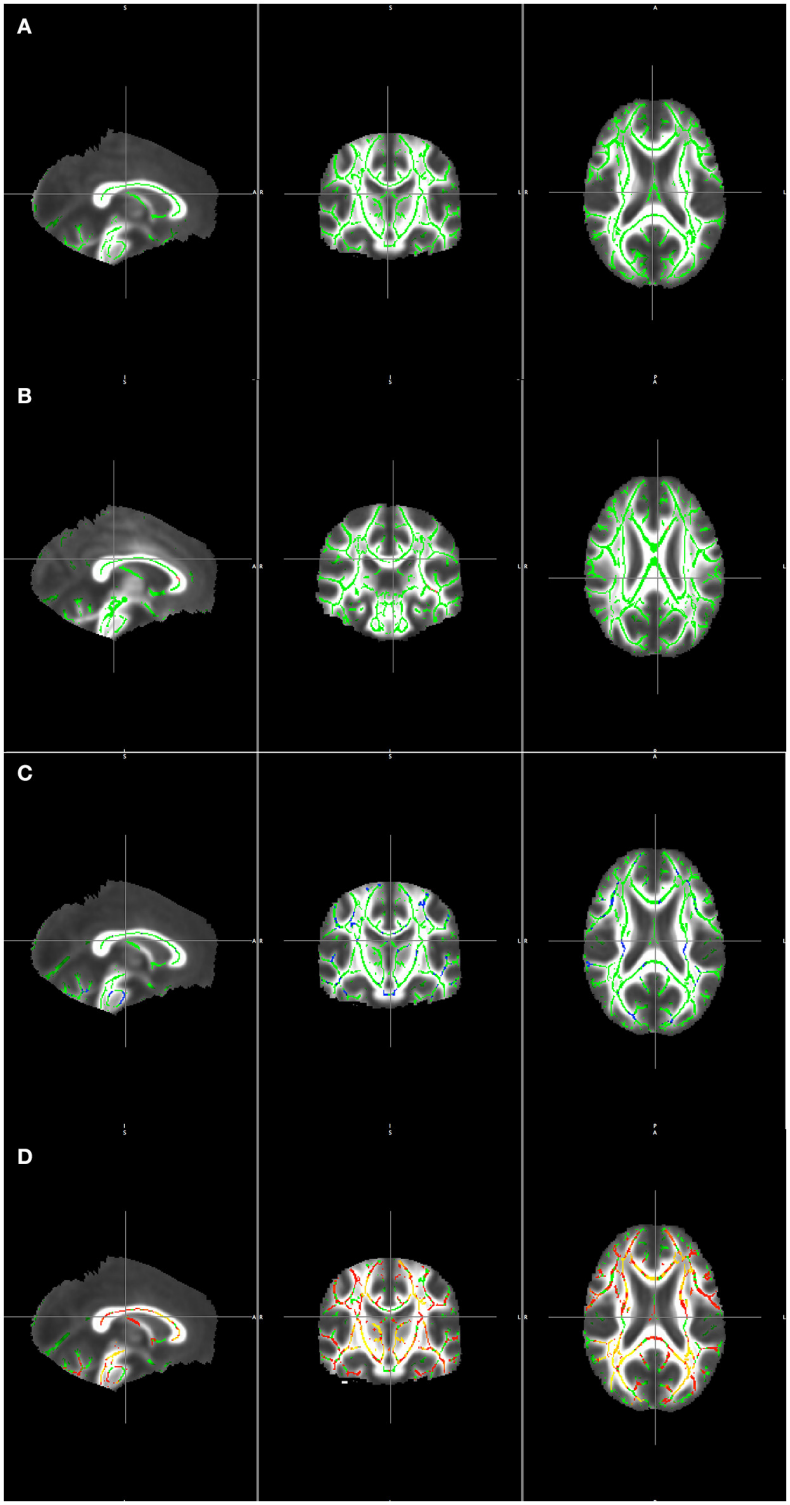
Visualization TBSS detection results masked by the *F*-test overlaid on the mean of registered FA volumes. **(A)** Mean skeleton (green). **(B)** F statistics (red-yelow) over the mean skeleton (green). **(C)** Clusters detected from contrast AD > LOBD masked by *F*-test clusters (blue) overlaying the mean skeleton (green). **(D)** Clusters detected from contrast LOBD > AD masked by *F*-test clusters (red) overlaying the mean skeleton (green).

Figure 2 illustrates the clusters detected by the AD > LOBD and LOBD > AD contrasts after masking with the *F*-test clusters. Figure 2A presents the mean FA volume and its skeleton (green). Figure 2B presents the *F*-test statistics (red). Figure 2C presents the significant clusters of the AD > LOBD contrast (blue). Figure 2D presents significant clusters of the LOBD > AD contrast.

**Figure 3:**
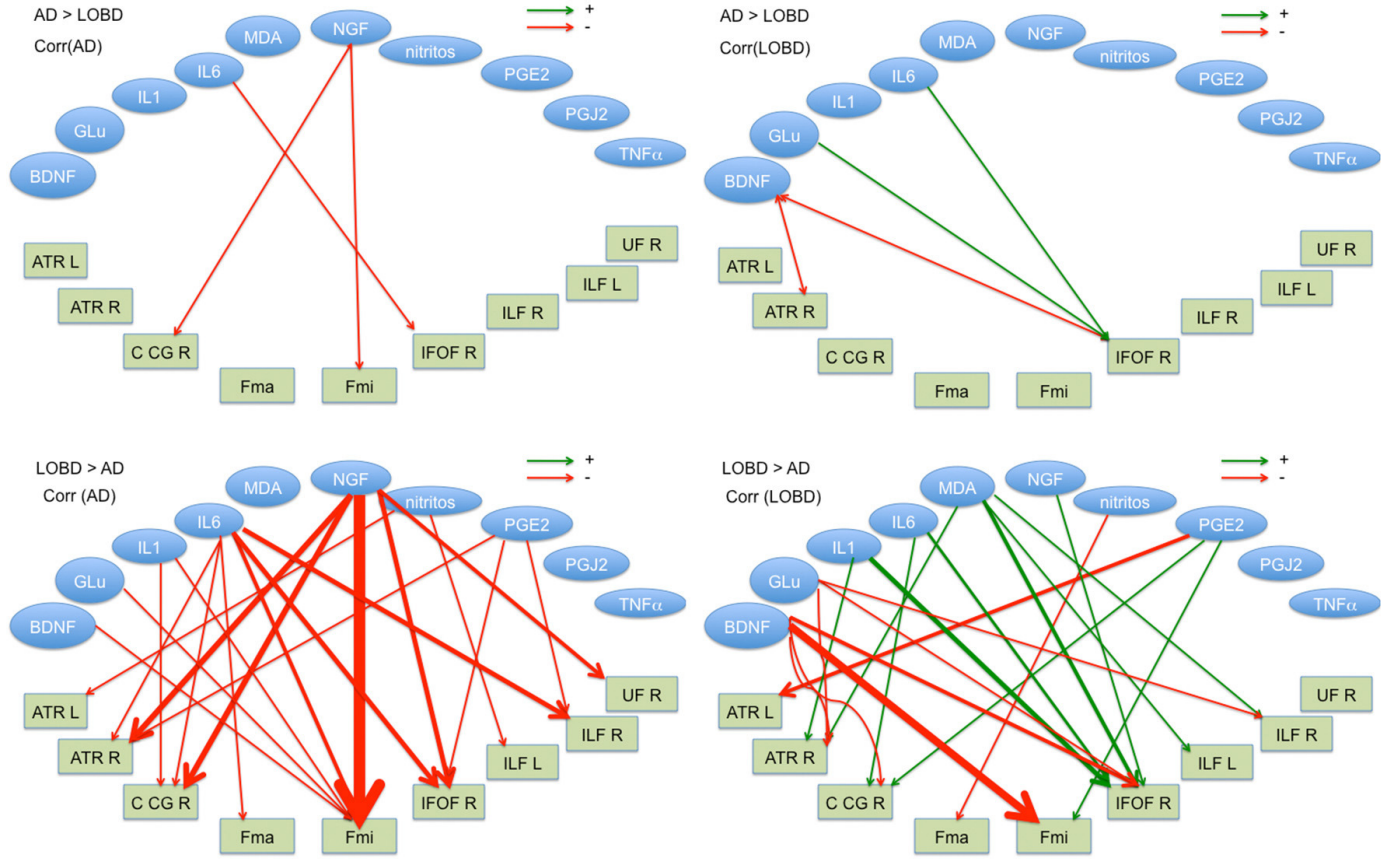
Significant correlation (*p* < 0.01) between blood plasma biomarkers (ellipses up) and FA values at the TBSS clusters for AD > LOBD (up) and LOBD > AD contrasts, masked by the *F*-test, identified by the atlasquery tool (rectangles below). We report separate values for AD and LOBD populations (requested by reviewer). Red lines correspond to negative correlations, green lines correspond to positive correlations. Line width is proportional to the magnitude of correlation. R, Right; L, left; ATR, hemispheres of anterior thalamic radiation; C_CG, cingulum (cingulate gyrus); FMi, forceps minor; FMa, forceps major; IFOF, inferior fronto-occipital fasciculus; ILF, inferior longitudinal fasciculus; SFL, superior longitudinal fasciculus; and UF, uncinate fasciculus.

Figure 3 shows in graphical form the correlation analysis results for each contrast of interest (AD > LOBD, LOBD > AD) and population (AD, LOBD). Nodes in the graph are either blood biomarkers (blue ellipsoids) or white matter tracts (rectangles), red arrows denote negative correlation, green arrows denote positive correlation. Table 1 gives the sizes of the clusters of positive and negative correlations. Notice that the AD > LOBD contrast effects are very small according to Figure 1. The strongest effects correspond to neurotrophic factors BDNF, and NGF, and inflammation marker IL6. The NGF accounts for 70% of all the correlation effects. BDNF appears positively correlated to LOBD and negatively correlated to AD.

**Table 1:** Size (#voxels) of the correlation clusters.

	**AD > LOBD**				**LOBD > AD**			
	**AD**		**LOBD**		**AD**		**LOBD**	
	+	−	+	−	+	−	+	−
BDNF				2		3		20
GLU	1					1		5
IL1β						5	9	
IL6		6	1			44	3	
MDA							8	
NGF		1				276	1	
NO2						2		2
PGE2						4	3	2
PGJ2								
TNFα								1

*Rows: blood biomarkers. Columns: for each contrast (AD > LOBD, LOBD > AD) and population (AD,LOBD), positive (+) and negative (−) correlation*.

## Discussion

We study differential effects in the WM tracts and their correlation to plasma biomarkers looking for new insights into the pathophysiological processes underlying AD and LOBD (Lebert et al., 2008; Carlino et al., 2013; Grande et al., 2014). We know that cognitive degradation in LOBD is a key factor in differential diagnosis between LOBD and AD. Besides cognitive performance, behavioral disorders are also closely related to the overall functionality of the patients. Agitation, euphoria and disinhibition are the non-cognitive neuropsychological variables having the greatest discrimination power in the classification of patients into AD or LOBD (Besga et al., 2015), while memory cognitive domain performance is essential in clinical practice for the detection and diagnosis of AD (Weintraub et al., 2012). Besides, recent studies have revealed that significant cognitive impairment in BD compared to controls may allow to discriminate type I and II BD patients (Aprahamian et al., 2014; Sparding et al., 2015), and may affect its prognosis, as it happens in patients with dementia (Kawas et al., 2003). Previously, some authors suggested that BD diagnosis is a significant predictor of long-term cognitive dysfunction increase (Lewandowski et al., 2011; Torrent et al., 2012). Although there are limited data on the cognitive profile of LOBD (Carlino et al., 2013; Grande et al., 2014), cognitive deficits affecting memory, attention and executive function have been reported for BD patients (Robinson et al., 2006; Osher et al., 2011; Aprahamian et al., 2014).

### TBSS localizations

We focus the discussion on the tracts where the *F*-test finds the greatest clusters for the LOBD > AD contrast, which has quantitatively greater effects than the AD > LOBD contrast. The size of cluster localizations suggest greater axonal degradation in AD than in LOBD in pathways that serve to integrate cognitive and social structures, including tracts that mediate connectivity to frontal and temporal lobes.

In particular, significant difference s found in ILF indicate degradation of the fronto-temporal-occipital circuit which is very important for social and emotional processing, leading to behavioral deterioration, which has been assessed as the main discriminant between AD and LOBD (Besga et al., 2015). The ILF tract connects the occipital cortex and temporal lobes including the superior, middle and anterior lobes, mediating the connectivity between three regions: the superior temporal sulcus, the fusiform face area, and the amygdala. Therefore, ILF degradation has impact on the processes of detecting biological motion and eye gaze (Pelphrey and Carter, 2008), as well as facial information processing with social significance, i.e., face identification and facial expression interpretation (Adolphs et al., 1999). We found also significant differences in the cingulate gyrus C_CG, which is part of the cingulate cortex lying above the corpus callosum, and part of the limbic system in charge of processing emotional contents. Together with the previously described impairments of ILF pathway, disruption of C_CG impedes the structural connectivity of an extended circuit that involves frontal, temporal and occipital regions. This circuit controls socio-emotional processing, so its degradation leads to greater behavioral an d emotional impairments of AD than LOBD.

The IFOF connects the occipital, posterior temporal, and the orbito-frontal areas (Ashtari, 2012). Simultaneously degraded axonal integrity of left UF, IFOF, and ATR has significant impact on the semantic processing (Han et al., 2013) during specific cognitive tasks related to object recognition. This concurrent effect can explain the increased cognitive impairment of the AD relative to LOBD (Besga et al., 2015), though we have not carried out correlation study of image data with cognitive neuropsychological tests results. The FMi collects most of the clusters detected, so its relative degradation in AD compared to LOBD is a salient biomarker. The FMi connects the lateral and medial surfaces of the frontal lobes crossing the midline via the genu of the corpus callosum. Damage of the FMi detected by decreasing FA in DTI imaging has been associated with fatigue and depression in multiple sclerosis (Gobbi et al., 2014). Degradation of FMiin mild cognitive impairment (MCI) and AD relative to HC was found in a cohort study using multiple diffussion measures (Alves et al., 2013).

### Correlation analysis

Peripheral biomarkers of inflammation, oxidative stress, and neurotrophins have been related to clinical symptoms, cognitive decline and illness severity in BD (Barbosa et al., 2012; Martinez-Cengotitabengoa et al., 2014) as well as in AD (Berridge, 2013). It has been suggested that inflammation and oxidative stress do not cause AD or LOBD by themselves, but that they reinforce interactions among factors related to these complex neuropsychiatric disorders during brain aging (Forcada et al., 2015), leading to a misbalance between protective and degenerative factors, which predisposes the brain to neurodegenerative diseases (Lewandowski et al., 2011). On the other hand, negative correlation of inflammation biomarkers, i.e., TNFα, with FA in the body and isthmus of the corpus callosum has been also found in healthy aging subjects by a TBSS analysis (Arfanakis et al., 2013), showing that systemic inflammation is not necessarily associated with cognitive decline. There are also reports of a significant decrease in BDNF and IL-6 in BD patients at a later stage compared to its early stage, while, inversely, TNFα has a significant increase at the later stage of BD (Kauer-Sant'Anna M, 2009; Grande et al., 2014), suggesting that the inflammation lies in the pathogenesis of BD.

Brain injuries promote the up-regulation of proinflamatory prostaglandins PGE2 (Ahmad et al., 2006), hence blocking the corresponding receptor has been proposed as a target of treatment for stroke and other traumatic brain injuries. Cyclopentenone Prostaglandin PGJ2 is a recently discovered prostaglandin, which has anti-inflammatory functions (Scher and Pillinger, 2005; Zhao et al., 2006), such as the inhibition of a gene in T cells, therefore positive correlation with FA voxels is consistently related to axonal integrity in this area. TNFα is a cytokine involved in systemic inflammation and acute phase reaction, whose role is the regulation of immune cells, inducing inflammation and other effects, such as apoptotic cell death.

The role of NGF as a therapeutic tool for AD has received a lot of attention in the last years (Xu et al., 2016), with strong consideration of the impairment of NGF pathway as cause of AD via the accumulation of amyloid plaques. Clinical trials have been carried out^3^^,^^4^ studying the effect of NGF gene therapy (Tuszynski et al., 2005). Postmortem analysis showed that the NGF treatment induced response in degenerating neurons exhibited trophic response without adverse pathological effects (Tuszynski et al., 2015).

In our study, regarding inflammation biomarkers in Table 1, we find no effect of the protective PGJ2, and almost no effect of TNFα, while there PGE2 has both positive and negative correlation with LOBD image data, so no conclusion can be given from them. However, though sparsely distributed in different clusters, we find positive correlation of IL1β and IL6 with LOBD and negative with AD, hence these blood biomarkers are a clear indication of greater inflammation in LOBD pathogenesis.

Regarding oxidative stress biomarkers, we found no differential effect of NO2, because both populations showed the same sizes of negative correlation clusters, but MDA shows positive correlation with LOBD hinting to an added pathogenesis factor. This result is also in agreement with our previous findings using eigenanatomy (Besga et al., 2016). Notice in Figure 3 that IL1β, IL6, and MAD affects mostly the IFOF as a cause for behavior impairment.

Regarding neurotrophic factors, we found a big effect of NGF which correlates negatively with AD imaging data, i.e., with the degradation of synaptic integrity in the located tract. We had also a small positive correlation with LOBD that reinforces the value of NGF as a differential diagnostic biomarker between AD and LOBD. This result is in complete agreement with recent AD therapeutic research lines (Tuszynski et al., 2005, 2015; Xu et al., 2016). Notice from Figure 3 that most of the correlation effects of NGF are located in FMi, suggesting a role in cognitive decline.

## Conclusions

TBSS analysis found widespread white matter disruption in LOBD relative to AD that might be related to axonal integrity degradation measured by decreasing FA in several important tracts. Main effects are located on white matter tracts that integrate a distributed fronto-temporal-occipital circuit. Disruption of this circuit may be producing the behavioral and cognitive impairments that differentiate LOBD from AD in the clinical and neuropsychological tests. Also, inter-hemispherical tracts FMihas greater axonal integrity degradation in AD than in LOBD, which is a pathophysiological cause for c ognitive decline of AD relative to LOBD. Finally, the correlation analysis suggests that neurotropic fact ors, i.e., NGF and BDNF, considered together with FA imaging may help to differentiate LOBD from AD. Also, there are indications of greater inflammation (IL1β,IL6) and oxidative stress (MDA) factors in LOBD than in AD.

## Author contributions

AB, IG, AG, and MG have made substantial contributions to the conception or design of the work; AB, DC, MG-L, and JE contributed to the acquisition, analysis, or interpretation of data for the work; all authors contributed drafting the work and revising it critically for important intellectual content; all authors give final approval of the version to be published; all authors agree to be accountable for all aspects of the work in ensuring that questions related to the accuracy or integrity of any part of the work are appropriately investigated and resolved.

### Conflict of interest statement

The authors declare that the research was conducted in the absence of any commercial or financial relationships that could be construed as a potential conflict of interest.

## Footnotes

^1^http://www.controlled-trials.com/search?q=HS%2FPI2010001

^2^http://fsl.fmrib.ox.ac.uk/fsl/fslwiki/TBSS

^3^https://clinicaltrials.gov/ct2/show/NCT00017940

^4^https://clinicaltrials.gov/ct2/show/NCT01163825

## References

[B1] Adolphs R. Tranel D. Hamann S. Young A. Calder A. E. (1999). Recognition of facial emotion in nine individuals with bilateral amygdala damage. Neuropsychologia 37, 1111–1117. 10.1016/S0028-3932(99)00039-1 10509833

[B2] Ahmad A. Saleem S. Ahmad M. Dore S. (2006). Prostaglandin ep1 receptor contributes to excitotoxicity and focal ischemic brain damage. Toxicol. Sci. 89, 265–270. 10.1093/toxsci/kfj022 16237196

[B3] Akiyama H. Barger S. Barnum S. Bradt B. Bauer J. Cole G.. (2000). Inflammation and Alzheimer's disease. Neurobiol. Aging 21, 383–421. 10.1016/S0197-4580(00)00124-X 10858586 PMC3887148

[B4] Albert M. Blacker D. (2006). Mild cognitive impairment and dementia. Annu. Rev. Clin. Psychol. 2, 379–388. 10.1146/annurev.clinpsy.1.102803.144039 17716075

[B5] Alves G. S. O'Dwyer L. Jurcoane A. Oertel-Knochel V. Knochel C. Prvulovic D.. (2013). Different patterns of white matter degeneration using multiple diffusion indices and volumetric data in mild cognitive impairment and Alzheimer patients. PLoS ONE 7:e52859. 10.1371/journal.pone.0052859 23300797 PMC3534120

[B6] Andersson J. Jenkinson M. Smith S. (2007a). Non-linear Optimisation. Technical Report TR07JA1, FMRIB.

[B7] Andersson J. Jenkinson M. Smith S. (2007b). Non-linear Registration, aka Spatial Normalisation. Technical Report TR07JA2, FMRIB.

[B8] Aprahamian I. Ladeira R. Diniz B. Forlenza O. Nunes P. (2014). Cognitive impairment in euthymic older adults with bipolar disorder: a controlled study using cognitive screening tests. Am. J. Geriatr. Psychiatry 22, 389–397. 10.1016/j.jagp.2012.08.013 23567429

[B9] Arfanakis K. Fleischman D. A. Grisot G. Barth C. M. Varentsova A. Morris M. C.. (2013). Systemic inflammation in non-demented elderly human subjects: brain microstructure and cognition. PLoS ONE 8:e73107. 10.1371/journal.pone.0073107 23991174 PMC3753267

[B10] Ashtari M. (2012). Anatomy and functional role of the inferior longitudinal fasciculus: a search that has just begun. Develop. Med. Child Neurol 54, 6–7. 10.1111/j.1469-8749.2011.04122.x 22098073

[B11] Avants B. Dhillon P. Kandel B. Cook P. McMillan C. Grossman M.. (2012). Eigenanatomy improves detection power for longitudinal cortical change. Med. Image Comput. Comput. Assist. Interv. 15, 206–213. 23286132 10.1007/978-3-642-33454-2_26 PMC3653970

[B12] Avants B. B. Libon D. J. Rascovsky K. Boller A. McMillan C. T. Massimo L.. (2014). Sparse canonical correlation analysis relates network-level atrophy to multivariate cognitive measures in a neurodegenerative population. Neuroimage 84, 698–711. 10.1016/j.neuroimage.2013.09.048 24096125 PMC3911786

[B13] Bach M. Laun F. B. Leemans A. Tax C. M. Biessels G. J. Stieltjes B.. (2014). Methodological considerations on tract-based spatial statistics (tbss). Neuroimage 100, 358–369. 10.1016/j.neuroimage.2014.06.021 24945661

[B14] Barbosa I. Rocha N. Huguet R. Ferreira R. Salgado J. Carvalho L. A. (2012). Executive dysfunction in euthymic bipolar disorder patients and its association with plasma biomarkers. J. Affect. Disord. 137, 151–155. 10.1016/j.jad.2011.12.034 22252095

[B15] Basser P. J. Mattiello J. LeBihan D. (1994). MR diffusion tensor spectroscopy and imaging. Biophys. J. 66, 259–267. 10.1016/S0006-3495(94)80775-1 8130344 PMC1275686

[B16] Bauer I. E. Pascoe M. Wollenhaupt-Aguiar B. Kapczinski F. Soares J. C. (2014). Inflammatory mediators of cognitive impairment in bipolar disorder. J. Psychiat. Res. 56, 18–27. 10.1016/j.jpsychires.2014.04.017 24862657 PMC4167370

[B17] Bauer M. Glenn T. Alda M. Andreassen O. Angelopoulos E. Ardau R.. (2015a). Influence of birth cohort on age of onset cluster analysis in bipolar i disorder. Euro. Psychiatry 30, 99–105. 10.1016/j.eurpsy.2014.10.005 25498240

[B18] Bauer M. Glenn T. Alda M. Andreassen O. A. Angelopoulos E. Ardau R.. (2015b). Influence of light exposure during early life on the age of onset of bipolar disorder. J. Psychiat. Res. 64, 1–8. 10.1016/j.jpsychires.2015.03.013 25862378

[B19] Bauer M. Glenn T. Alda M. Andreassen O. A. Angelopoulos E. Ardau R.. (2014). Relationship between sunlight and the age of onset of bipolar disorder: an international multisite study. J. Affect. Disord. 167, 104–111. 10.1016/j.jad.2014.05.032 24953482

[B20] Behrens T. E. Woolrich M. W. Jenkinson M. Johansen-Berg H. Nunes R. G. Clare S.. (2003). Characterization and propagation of uncertainty in diffusion-weighted MR imaging. Magn. Reson. Med. 50, 1077–1088. 10.1002/mrm.10609 14587019

[B21] Berlyand Y. Weintraub D. Xie S. X. Mellis I. A. Doshi J. Rick J.. (2016). An Alzheimer's disease-derived biomarker signature identifies parkinson's disease patients with dementia. PLoS ONE 11:e0147319. 10.1371/journal.pone.0147319 26812251 PMC4727929

[B22] Besga A. Chyzhyk D. Gonzalez-Ortega I. Savio A. Ayerdi B. Echeveste J.. (2016). Eigenanatomy on fractional anisotropy imaging provides white matter anatomical features discriminating between Alzheimer's disease and late onset bipolar disorder. Curr. Alzheimer Res. 13, 557–565. 10.2174/1567205013666151116125349 26567744

[B23] Besga A. Gonzalez-Ortega I. Echeburua E. Savio A. Ayerdi B. Chyzhyk D.. (2015). Discrimination between Alzheimer's disease and late onset bipolar disorder using multivariate analysis. Front. Aging Neurosci. 7:231. 10.3389/fnagi.2015.00231 26696883 PMC4677464

[B24] Besga A. Martinez-Cengotitabengoa M. Gonzalez-Ortega I. Gutierrez M. Barbeito S. Gonzalez-Pinto A. (2011). The role of white matter damage in late onset bipolar disorder. Maturitas 70, 160–163. 10.1016/j.maturitas.2011.07.005 21872409

[B25] Besga A. Termenon M. Graña M. Echeveste J. Perez J. Gonzalez-Pinto A. (2012). Discovering Alzheimer's disease and bipolar disorder white matter effects building computer aided diagnostic systems on brain diffusion tensor imaging features. Neurosci. Lett. 520, 71–76. 10.1016/j.neulet.2012.05.033 22617636

[B26] Brydon L. Walker C. Wawrzyniak A. Whitehead D. Okamura H. Yajima J.. (2009). Synergistic effects of psychological and immune stressors on inflammatory cytokine and sickness responses in humans. Brain Behav. Immun. 23, 217–224. 10.1016/j.bbi.2008.09.007 18835437 PMC2637301

[B27] Carlino A. Stinnett J. Kim D. (2013). New onset of bipolar disorder in late life. Psychosomatics 54, 94–97. 10.1016/j.psym.2012.01.006 22652303 PMC3914401

[B28] Castanon N. Lasselin J. Capuron L. (2014). Neuropsychiatric comorbidity in obesity: role of inflammatory processes. Front. Endocrinol (Lausanne). 5:74. 10.3389/fendo.2014.00074 24860551 PMC4030152

[B29] Chou P.-H. Tseng W.-J. Chen L.-M. Lin C.-C. Lan T.-H. Chan C.-H. (2015). Late onset bipolar disorder: a case report and review of the literature. J. Clin. Gerontol. Geriatr. 6, 27–29. 10.1016/j.jcgg.2014.05.002

[B30] Depp C. Jeste D. (2004). Bipolar disorder in older adults: a critical review. Bipolar Disord. 6, 343–367. 10.1111/j.1399-5618.2004.00139.x 15383127

[B31] Dickerson F. Stallings C. Origoni A. Vaughan C. Katsafanas E. Khushalani S.. (2013). A combined marker of inflammation in individuals with mania. PLoS ONE 8:e73520. 10.1371/journal.pone.0073520 24019926 PMC3760815

[B32] Forcada I. Mur M. Mora E. Vieta E. Bartras-Faz D. Portella M. J. (2015). The influence of cognitive reserve on psychosocial and neuropsychological functioning in bipolar disorder. Euro. Neuropsychopharmacol. 25, 214–222. 10.1016/j.euroneuro.2014.07.018 25172270

[B33] Garcia-Bueno M. Bioque M. Mac-Dowell K. S. Barcones M. F. Martínez-Cengotitabengoa M. Pina-Camacho L.. (2014). Proanti-inflammatory dysregulation in patients with first episode of psychosis: toward an integrative inflammatory hypothesis of schizophrenia. Schizophr. Bull. 40, 376–387. 10.1093/schbul/sbt001 23486748 PMC3932081

[B34] Gobbi C. Rocca M. Pagani E. Riccitelli G. Pravatà E. Radaelli M.. (2014). Forceps minor damage and co-occurrence of depression and fatigue in multiple sclerosis. Multiple Scleros. J. 20, 1633–1640. 10.1177/1352458514530022 24740370

[B35] Goldstein B. Kemp D. Soczynska J. McIntyre R. (2009). Inflammation and the phenomenology, pathophysiology, comorbidity, and treatment of bipolar disorder: a systematic review of the literature. J. Clin. Psychiatry 70, 1078–1090. 10.4088/JCP.08r04505 19497250

[B36] Graña M. Termenon M. Savio A. Gonzalez-Pinto A. Echeveste J. Perez J.. (2011). Computer aided diagnosis system for Alzheimer disease using brain diffusion tensor imaging features selected by pearson's correlation. Neurosci. Lett. 502, 225–229. 10.1016/j.neulet.2011.07.049 21839143

[B37] Grande I. Magalhaes P. Chendo I. Stertz L. Panizutti B. Colpo G.. (2014). Staging bipolar disorder: clinical, biochemical, and functional correlates. Acta Psychiatr. Scand. 129, 437–444. 10.1111/acps.12268 24628576

[B38] Han Z. Ma Y. Gong G. He Y. Caramazza A. Bi Y. (2013). White matter structural connectivity underlying semantic processing: evidence from brain damaged patients. Brain 136, 2952–2965. 10.1093/brain/awt205 23975453

[B39] Hope S. Hoseth E. Dieset I. Mørch R. H. Aas M. Aukrust P.. (2015). Inflammatory markers are associated with general cognitive abilities in schizophrenia and bipolar disorder patients and healthy controls. Schizophr. Res. 165, 188–194. 10.1016/j.schres.2015.04.004 25956633

[B40] Kamer A. Craig R. Dasanayake A. Brys M. Glodzik-Sobanska L. de Leon M. (2008). Inflammation and Alzheimer's disease: possible role of periodontal diseases. Alzheimer Dement. 4, 242–250. 10.1016/j.jalz.2007.08.004 18631974

[B41] Kauer-Sant'Anna M. Kapczinski F. Andreazza A. C. Bond D. J. Lam R. W. Young L. T.. (2009). Brain-derived neurotrophic factor and inflammatory markers in patients with early- vs. late-stage bipolar disorder. Int. J. Neuropsychopharmacol. 12, 447–458. 10.1017/S1461145708009310 18771602

[B42] Kawas C. Corrada M. Brookmeyer R. Morrison A. Resnick S. Zonderman A.. (2003). Visual memory predicts Alzheimer's disease more than a decade before diagnosis. Neurology 60, 1089–1093. 10.1212/01.WNL.0000055813.36504.BF 12682311

[B43] Kendall M. G. Stuart A. (1973). The Advanced Theory of Statistics, Volume 2: Inference and Relationship. London: Griffin.

[B44] Konradi C. Sillivan S. Clay H. (2012). Mitochondria, oligodendrocytes and inflammation in bipolar disorder: evidence from transcriptome studies points to intriguing parallels with multiple sclerosis. Neurobiol. Dis. 45, 37–47. 10.1016/j.nbd.2011.01.025 21310238 PMC3117935

[B45] Lebert F. Lys H. Haem E. Pasquier F. (2008). Dementia following bipolar disorder. Encephale 34, 606–610. 10.1016/j.encep.2007.12.007 19081458

[B46] Leboyer M. Soreca I. Scott J. Frye M. Henry C. Tamouza R.. (2012). Can bipolar disorder be viewed as a multi-system inflammatory disease? J. Affect. Disord. 141, 1–10. 10.1016/j.jad.2011.12.049 22497876 PMC3498820

[B47] Lee S.-Y. Chen S.-L. Chang Y.-H. Chen P. Huang S.-Y. (2013). Inflammation's association with metabolic profiles before and after a twelve-week clinical trial in drug-naive patients with bipolar ii disorder. PLoS ONE 8:e66847. 10.1371/journal.pone.0066847 23826157 PMC3695222

[B48] Lewandowski K. Cohen B. Ongur D. (2011). Evolution of neuropsychological dysfunction during the course of schizophrenia and bipolar disorder. Psychol. Med. 41, 225–241. 10.1017/S0033291710001042 20836900

[B49] Martinez-Cengotitabengoa M. Mico J. Arango C. Castro-Fornieles J. Graell M. Paya B.. (2014). Basal low antioxidant capacity correlates with cognitive deficits in early onset psychosis. a 2-year follow-up study. Schizophr. Res. 156, 23–29. 10.1016/j.schres.2014.03.025 24768133

[B50] Tuszynski M. H. Yang J. H. Barba D. U H. S. Bakay R. A. Pay M. M.. (2015). Nerve growth factor gene therapy: activation of neuronal responses in Alzheimer disease. JAMA Neurol. 72, 1139–1147. 10.1001/jamaneurol.2015.1807 26302439 PMC4944824

[B51] Berridge M. J. (2013). Dysregulation of neural calcium signaling in Alzheimer disease, bipolar disorder and schizophrenia. Prion 7, 2–13. 10.4161/pri.21767 22895098 PMC3609045

[B52] Osher Y. Dobron A. Belmaker R. Bersudsky Y. Dwolatzky T. (2011). Computerized testing of neurocognitive function in euthymic bipolar patients compared to those with mild cognitive impairment and cognitively healthy controls. Psychother. Psychosomat. 80, 298–303. 10.1159/000324508 21646824

[B53] Pearson K. (1895). Notes on regression and inheritance in the case of two parents. Proc. R. Soc. Lond. 58, 240–242. 10.1098/rspl.1895.0041

[B54] Pelphrey K. Carter E. (2008). Charting the typical and atypical development of the social brain. Dev. Psychopathol. 20, 1081–1102. 10.1017/S0954579408000515 18838032

[B55] Pierpaoli C. Jezzard P. Basser P. J. Barnett A. Chiro G. D. (1996). Diffusion tensor MR imaging of the human brain. Radiology 201, 637–648. 10.1148/radiology.201.3.8939209 8939209

[B56] Prabhakar D. Balon R. (2010). Late-onset bipolar disorder a case for careful appraisal. Psychiatry (Edgmont) 7, 34–37. 20386635 PMC2848458

[B57] Ramirez J. Gorriz J. Segovia F. Chaves R. Salas-Gonzalez D. Lopez M. (2010). Early Alzheimer's disease diagnosis using partial least squares and random forests, in Biomedical Imaging: From Nano to Macro, 2010 IEEE International Symposium on (Rotterdam), 81–84.

[B58] Robinson L. Thompson J. Gallagher P. Goswami U. Young A. Ferrier I. N.. (2006). A meta-analysis of cognitive deficits in euthymic patients with bipolar disorder. J. Affect. Disord. 93, 105–115. 10.1016/j.jad.2006.02.016 16677713

[B59] Salas-Gonzalez D. Górriz J. M. Ramírez J. López M. Illan I. A. Segovia F.. (2009). Analysis of SPECT brain images for the diagnosis of Alzheimer's disease using moments and support vector machines. Neurosci. Lett. 461, 60–64. 10.1016/j.neulet.2009.05.056 19477227

[B60] Sardi F. Fassina L. Venturini L. Inguscio M. Guerriero F. Rolfo E.. (2011). Alzheimer's disease, autoimmunity and inflammation. the good, the bad and the ugly. Autoimmun. Rev. 11, 149–153. 10.1016/j.autrev.2011.09.005 21996556

[B61] Savio A. Garcia-Sebastian M. Chyzyk D. Hernandez C. Graña M. Sistiaga A.. (2011). Neurocognitive disorder detection based on feature vectors extracted from vbm analysis of structural mri. Comput. Biol. Med. 41, 600–610. 10.1016/j.compbiomed.2011.05.010 21621760

[B62] Scher J. U. Pillinger M. H. (2005). 15d-pgj2: The anti-inflammatory prostaglandin? Clin. Immunol. 114, 100–109. 10.1016/j.clim.2004.09.008 15639643

[B63] Sigut J. Piñeiro J. Gonzalez E. Torres J. (2007). An expert system for supervised classifier design: application to Alzheimer diagnosis. Expert Syst. Applic. 32, 927–938. 10.1016/j.eswa.2006.01.026

[B64] Singhal G. Jaehne E. Corrigan F. Toben C. Baune B. (2014). Inflammasomes in neuroinflammation and changes in brain function: a focused review. Front. Neurosci. 8:315. 10.3389/fnins.2014.00315 25339862 PMC4188030

[B65] Smith S. M. Jenkinson M. Johansen-Berg H. Rueckert D. Nichols T. E. Mackay C. E.. (2006). Tract-based spatial statistics: Voxelwise analysis of multi-subject diffusion data. Neuroimage 31, 1487–1505. 10.1016/j.neuroimage.2006.02.024 16624579

[B66] Smith S. M. Jenkinson M. Woolrich M. W. Beckmann C. F. Behrens T. E. Johansen-Berg H.. (2004). Advances in functional and structural MR image analysis and implementation as FSL. Neuroimage, 23(Suppl. 1), S208–S219. Mathematics in Brain Imaging. 10.1016/j.neuroimage.2004.07.051 15501092

[B67] Smith S. M. Nichols T. E. (2009). Threshold-free cluster enhancement: addressing problems of smoothing, threshold dependence and localisation in cluster inference. Neuroimage 44, 83–98. 10.1016/j.neuroimage.2008.03.061 18501637

[B68] Sparding T. Silander K. Palsson E. Ostlind J. Sellgren C. Ekman C. J.. (2015). Cognitive functioning in clinically stable patients with bipolar disorder i and ii. PLoS ONE 10:e0115562. 10.1371/journal.pone.0115562 25614986 PMC4304812

[B69] Termenon M. Graña M. Besga A. Echeveste J. Gonzalez-Pinto A. (2013). Lattice independent component analysis feature selection on diffusion weighted imaging for Alzheimer's disease classification. Neurocomputing 114, 132–141. 10.1016/j.neucom.2012.08.044

[B70] Torrent C. Martinez-Aran A. del Mar Bonnin C. Reinares M. Daban C. Sole B.. (2012). Long-term outcome of cognitive impairment in bipolar disorder. J. Clin. Psychiatry 73, e899–e905. 10.4088/JCP.11m07471 22901360

[B71] Tuszynski M. H. Thal L. Pay M. Salmon D. P. U H. S. Bakay R.. (2005). A phase 1 clinical trial of nerve growth factor gene therapy for Alzheimer disease. Nat. Med. 11, 551–555. 10.1038/nm1239 15852017

[B72] Weintraub S. Wicklund A. Salmon D. (2012). The neuropsychological profile of Alzheimer disease. Cold Spring Harb. Perspect. Med. 2:a006171. 10.1101/cshperspect.a006171 22474609 PMC3312395

[B73] Westman E. Simmons A. Zhang Y. Muehlboeck J. Tunnard C. Liu Y.. (2011). Multivariate analysis of mri data for Alzheimer's disease, mild cognitive impairment and healthy controls. Neuroimage 54, 1178–1187. 10.1016/j.neuroimage.2010.08.044 20800095

[B74] Xu C.-J. Wang J.-L. Jin W.-L. (2016). The emerging therapeutic role of ngf in Alzheimer's disease. Neurochem. Res. 41, 1211–1218. 10.1007/s11064-016-1829-9 26801170

[B75] Zahodne L. B. Ornstein K. Cosentino S. Devanand D. Stern Y. (2015). Longitudinal relationships between Alzheimer disease progression and psychosis, depressed mood, and agitation/aggression. Am. J. Geriat. Psychiatry 23, 130–140. 10.1016/j.jagp.2013.03.014 23871118 PMC3858495

[B76] Zanetti M. V. Cordeiro Q. Busatto G. F. (2007). Late onset bipolar disorder associated with white matter hyperintensities: a pathophysiological hypothesis. Progr. Neuro Psychopharmacol. Biol. Psychiatry 31, 551–556. 17107742 10.1016/j.pnpbp.2006.10.004

[B77] Zhao X. Zhang Y. Strong R. Grotta J. C. Aronowski J. (2006). 15d-prostaglandin j2 activates peroxisome proliferator-activated receptor-γ, promotes expression of catalase, and reduces inflammation, behavioral dysfunction, and neuronal loss after intracerebral hemorrhage in rats. J. Cereb. Blood Flow Metab. 26, 811–820. 10.1038/sj.jcbfm.9600233 16208315

